# Risk Factors for Urgency Incontinence in Women Undergoing Stress Urinary Incontinence Surgery

**DOI:** 10.1155/2013/567375

**Published:** 2013-11-07

**Authors:** Leslie M. Rickey, Liyuan Huang, David D. Rahn, Yvonne Hsu, Heather J. Litman, Elizabeth R. Mueller

**Affiliations:** ^1^Departments of Urology and Obstetrics, Gynecology & Reproductive Sciences, Yale School of Medicine, Female Pelvic Medicine & Reconstructive Surgery, Yale-New Haven Hospital, Yale University, 310 Cedar Street, FMB 329E, New Haven, CT 06519, USA; ^2^New England Research Institute, Watertown, MA, USA; ^3^University of Texas Southwestern Medical Center, Dallas, TX, USA; ^4^University of Utah, Salt Lake City, UT, USA; ^5^Boston Children's Hospital, Boston, MA, USA; ^6^Loyola University Medical Center, Maywood, IL, USA

## Abstract

*Objective.* To determine baseline variables associated with urgency urinary incontinence (UUI) in women presenting for stress urinary incontinence (SUI) surgery. *Methods*. Baseline data from two randomized trials enrolling 1,252 women were analyzed: SISTEr (fascial sling versus Burch colposuspension) and TOMUS (retropubic versus transobturator midurethral sling). Demographic data, POP-Q measures, and validated measures of symptom severity and quality of life were collected. Charlson Comorbidity Index (CCI) and Patient Health Questionnaire-9 were measured in TOMUS. Multivariate models were constructed with UUI and symptom severity as outcomes. *Results*. Over two-thirds of subjects reported bothersome UUI at baseline. TOMUS patients with more comorbidities had higher UDI irritative scores (CCI score 0 = 39.4, CCI score 1 = 42.1, and CCI score 2+ = 51.0, *P* = 0.0003), and higher depression scores were associated with more severe UUI. Smoking, parity, prior incontinence surgery/treatment, prolapse stage, and incontinence episode frequency were not independently associated with UUI. *Conclusions*. There were no modifiable risk factors identified for patient-reported UUI in women presenting for SUI surgery. However, the direct relationships between comorbidity level, depression, and worsening of UUI/urgency symptoms may represent targets for preoperative intervention. Further research is necessary to elucidate the pathophysiologic mechanisms that explain the associations between these medical conditions and bladder function.

## 1. Introduction

The presence of stress urinary incontinence (SUI) or urgency urinary incontinence (UUI) has profound negative impacts on quality of life, exceeding that of other common morbid conditions such as diabetes [1]. However, women with *both* SUI and UUI—that is, mixed urinary incontinence (MUI)—have greater bother than those with SUI alone [2] and are less likely to have success or cure after undergoing surgical management of their SUI [3]. While multiple risk factors for worsening incontinence have been identified including age, parity, and body mass index [4], there is less known on whether other demographic variables, concurrent prolapse, or medical comorbidities may be associated with UUI in women presenting for surgical treatment of SUI. Better characterization of these associated variables may identify targets for intervention to improve outcomes and quality of life.

This study is an ancillary analysis employing the databases of two large randomized trials of women presenting for surgical management of SUI: the Trial of Mid-Urethral Slings (TOMUS) study [5] and the Stress Incontinence Surgical Treatment Efficacy Trial (SISTEr) [6]. Both studies had large numbers of patients with MUI. The primary objective of this study is to determine whether demographic factors, altered pelvic floor anatomy, or other comorbidities are associated with UUI among these women presenting for surgical management of SUI. A secondary goal is to better characterize the relationships between urgency symptoms and overall urinary symptom level, anatomic support, and quality of life measures.

## 2. Materials and Methods

### 2.1. Participants

 We analyzed baseline data from two IRB-approved randomized surgical trials for women undergoing SUI surgery conducted by the Urinary Incontinence Treatment Network (UITN). All participants provided written informed consent. SISTEr randomized 655 women with predominant SUI to either an autologous rectus fascial sling or the Burch colposuspension [6]. The UITN Trial of Mid-Urethral Slings (TOMUS) study randomized 597 women to retropubic versus transobturator midurethral slings for SUI [5]. For both studies, eligibility criteria included a Medical, Epidemiologic, and Social Aspects of Aging questionnaire (MESA) stress incontinence symptom score greater than MESA urge incontinence symptom score and confirmed observation of leakage by cough and Valsalva stress test at a standardized bladder volume of 300 mL. The SISTEr trial allowed concomitant abdominal surgery, whereas the TOMUS trial did not. Both trials allowed concomitant vaginal surgeries. Women who participated in the SISTEr trial or who had a prior synthetic sling for SUI or synthetic mesh which is placed for vaginal reconstructive surgery were excluded from the TOMUS trial. 

### 2.2. Measures

Baseline demographic data included age, self-reported race/ethnicity, and education. Body mass index (BMI, recorded in kg/m^2^) was calculated from measured height and weight. Specific factors which are related to obstetrical and gynecological history included the number of pregnancies (categorized as 0, 1, 2, and ≥3), menopausal status, hormone replacement therapy, prior hysterectomy, and prior surgery and/or treatment for urinary incontinence or pelvic organ prolapse. 

### 2.3. Questionnaires

Subjective urinary incontinence symptoms were measured using validated questionnaires: Urogenital Distress Inventory (UDI) [7], Incontinence Impact Questionnaire (IIQ) [7], MESA questionnaire [8], and the Patient Global Impression of Severity (PGI-S) scale [9]. 

The UDI measures 3 domains: stress incontinence symptoms, irritative symptoms, and obstructive/discomfort symptoms with bother being measured with a 4-point Likert scale. The scores from each domain are transformed into a continuous scale ranging from 0 to 100, and higher scores indicate greater bother. For the purpose of this analysis, urgency urinary incontinence was defined as present if a subject reported urinary leakage related to the feeling of urgency as “moderate or greatly bothersome” on the UDI. In addition, the irritative domain was used as a measure of a woman's urge symptoms.

The IIQ consists of 30 items that query the impact of urogenital symptoms on physical functioning, emotional functioning, travel/mobility, and social functioning using a 4-point Likert scale. Each domain is transformed into a scale ranging from 0 to 100 with higher values representing more negative impact. 

The MESA questionnaire consists of 15 items total divided into stress and urge subscales. A higher MESA score indicates more frequent symptoms overall (range of 0–45) and for each incontinence subscale (range of 0–27 for SUI and range of 0–18 for UUI).

The following 3 questionnaires were used only in the TOMUS trial. The PGI-S questionnaire asks subjects to describe “how their urinary condition is now” and consists of 4 choices for response: normal, mild, moderate, and severe. The Charlson Comorbidity Index (CCI) encompasses 19 medical conditions weighted from 1 to 6 with total scores ranging from 0 to 37. A CCI of 3-4 is associated with 52% 1-year mortality [10]. The Patient Health Questionnaire-9 (PHQ-9) was developed for screening, diagnosing, and assessing the severity of a depressive disorder. The summary score ranges from 0 to 27, and a higher score indicates more depressive symptoms. A validation study of the PHQ-9 showed that a score of 10 appeared to discriminate between those with no depressive disorder (<10) and those with major depression (≥10) [11].

### 2.4. Objective Measures

Objective measures included a 24-hour pad test, POP-Q measurements, and bladder volume at the time of a positive stress test. 

### 2.5. Statistical Methods

Analyses were carried out in parallel for the SISTEr and TOMUS subjects as the trials had different inclusion and exclusion criteria representing different populations. Bivariate logistic regression models were fit considering each covariate separately with UUI as the outcome (UUI is defined as present if a subject reported urine leakage related to the feeling of urgency as moderately/greatly bothersome on UDI). Covariates that were statistically and significantly (*P* < 0.05) associated with the odds of UUI in SISTEr or in TOMUS were considered in the multivariable model. A final multivariable model was fit to include all the common covariates from preliminary multivariable models that were significantly associated with the outcome in either trial. Urinary incontinence severity (PGI-S), depression, and the number of comorbidities were measured only in TOMUS; these variables were included in the final TOMUS models as appropriate. Odds ratios (ORs) with corresponding 95% confidence intervals (CI) were reported.

Linear regression modeling was used to identify associations with irritative/urgency symptoms reported on the UDI and MESA. A similar, yet independent, technique was used for building the linear regression multivariable models. Specifically, covariates that were statistically and significantly associated with irritative/urgency symptoms were considered in the multivariable models. For continuous explanatory variables, the slope coefficient for the association between a particular independent covariate and UDI irritative subscale/MESA urge symptoms controlling for the other covariates in the model was presented. For categorical covariates, the adjusted mean UDI irritative subscale/MESA urge symptoms controlling for the other covariates in the model was reported for each level of the categorical variable.

All statistical analysis was performed using SAS version 9.2 (SAS Institute, Inc, Cary, NC). A 5% two-sided significance level was used for all statistical testing.

## 3. Results

Over two-thirds of women with stress predominant incontinence in both studies reported being moderately or greatly bothered by urine leakage related to the feeling of urgency (UUI in 70% in SISTEr and 68% in TOMUS). This is also reflected in the relatively high mean (standard deviation) UDI-I (47.8 (25.2) in SISTEr and 41.2 (25.4) in TOMUS) and MESA urge scores (6.3 (4.0) in SISTEr and 6.2 (4.0) in TOMUS). The descriptive statistics for the two trials based on the presence or absence of UUI are shown in Table 1. Bivariate analysis revealed that menopausal and hormone status, education attainment, MESA stress score, BMI, and IIQ were associated with greater odds of UUI in both studies (Table 1). It should be noted that a significant association was not found between UUI and race/ethnicity, parity, smoking, or prolapse stage. After multivariate analysis, only IIQ score was found to be independently associated with the presence of UUI. For every 10-point increase in IIQ score (indicating worse quality of life), there was a 7% increase in the prevalence of UUI (*P* < 0.0001) in both study populations.

Multivariate analyses were carried out using the continuous variables of UDI-I (Table 2) and MESA urge scores (Table 3) to measure bother and severity of LUTS (including UUI) as an outcome compared to the dichotomous nature of primary definition of UUI. Race/ethnicity was found to be significantly associated with UDI-I score in SISTEr but not in TOMUS (Table 2). For both study populations, the UDI-I and MESA urge scores were found to differ according to education achievement, and those subjects with less than high school education tended to have the highest (or most bothersome) scores. In TOMUS and SISTEr, pad weight was independently associated with the severity of UDI-I and MESA urge; however, the increases in score were small, so this finding may not be clinically significant. In TOMUS subjects, higher depression PHQ-9 scores and increasing number and/or severity of comorbidities (CCI) were associated with more severe UUI/urgency symptoms. Age was also independently associated with greater UUI severity. For every 10-year increase in age, there was an approximately 0.5-point increase in MESA urge score. Greater severity (PGI-S) and bother from stress incontinence on UDI (UDI-S) and MESA (MESA stress) were associated with worsening UUI and irritative symptoms.

## 4. Discussion

 Richter et al. demonstrated that higher MESA urge scores were associated with increased odds of failure after SUI surgery [3], and in a retrospective review of 464 women, the presence of UUI was an independent risk factor for failure after mid-urethral sling [12]. Therefore, we sought to determine baseline variables associated with UUI and identify any modifiable factors that could potentially improve SUI surgery outcomes. Although we did find that quality of life was negatively impacted by the presence of UUI in both patient populations, we were not able to identify other patient characteristics that were associated with UUI. However, when a definition that included a broader range of irritative LUTS was used, the presence of depression and more comorbidities were associated with greater bother and severity of urge-related symptoms in TOMUS subjects, independent of potential confounders such as age and SUI severity. 

 Urgency urinary incontinence commonly coexists in women undergoing surgery for SUI. More than two-thirds of the 1252 subjects from the SISTEr and TOMUS studies reported “moderately or greatly bothersome” UUI at baseline. We found no modifiable patient characteristics that are associated with the presence of preoperative bothersome UUI in this population of women. However, patients with more comorbidities reported greater bother from LUTS, and higher depression scores were associated with more severe UUI symptoms. Women with UUI reported worse incontinence-related QoL compared to women without UUI, and as bother from irritative LUTS increased, QoL was negatively impacted. Interestingly, similar to the findings of Lowder et al. [13], the severity of SUI symptoms predicted greater UUI symptoms as well as more bother from overall irritative LUTS. It has also been shown that overall incontinence severity as measured by incontinence episodes or patient impression of severity is associated with concurrent urge symptoms in women with SUI [14]. While urgency and frequency may result from adaptive behaviors to prevent stress-related leakage, the relationship between SUI and UUI could represent either greater sphincter dysfunction or more widespread neuromuscular dysfunction of the pelvic floor.

 Age and BMI have been the most consistently reported risk factors associated with UUI in large studies of community dwelling women [15, 16] and were also found to be risk factors in a twin study [17]. However, most of those studies are analyses of population surveys and do not include POP-Q measurements, voiding diary, or quality of life data. In the current study, parity, prior UI surgery/treatment, POP-Q stage, hormonal status, and incontinence episode frequency were not independently associated with UUI or bother from UUI and LUTS as measured by the UDI-I and MESA urge. Our findings that vaginal anatomy and support were not related to LUTS are consistent with previous studies [18, 19]. Although parity has been reported as a risk factor for urgency and UUI [17, 20]; when subjects with pure or predominant UUI are studied, parity tends to show an association with SUI and/or MUI, but not with UUI [15, 16, 21]. 

 Associations between depression and urinary incontinence have been identified in large population-based cross-sectional studies [22, 23] as well as prospective cohort studies [21, 24]. Depressive symptoms were more likely to be reported by women with incontinence compared to continent women [22], and in a population of subjects greater than 70 years old, depressive mood was a predictor of incident UUI over a 6-year period [24]. Although it is possible that incontinence as a chronic disease may lead to depression, a recent study by Melville et al. [25] suggests that major depression may lead to increased odds of incident incontinence. The investigators found that subjects with depression were more likely to develop incontinence over 6 years (OR 1.46, 1.08–1.97), whereas baseline incontinence did not predict onset of depression (OR 1.03, 0.75–1.42). Further, there is basic science evidence that common biochemical pathways involving neurotransmitters such as serotonin [26] and corticotropin releasing factor [27] may link the two conditions. Alterations in these neurotransmitters have been associated with inducing overactive bladder-type symptoms in animal models. In the few population studies that have differentiated groups based on type of incontinence, it appears that subjects with depressive mood may be more likely to report UUI or MUI compared to SUI [21, 22]. Likewise, although the difference was relatively small, we also demonstrated that higher depression scores were associated with more severe UUI as measured by the MESA urge subscale. Continued basic science and translational research in this area may lead to therapeutic targets as well as further clarification of the central and peripheral neural alterations that lead to overactive bladder symptoms. 

 The effect of concomitant medical conditions on UUI and LUTS was evaluated using the CCI, and as the CCI score increased, women reported significantly more bother from LUTS. Similar findings were reported by Nuotio et al., who found that older patients with ≥4 chronic diseases had more than 5 times the risk of developing new UUI (CI 2.03–15.54) compared to those with fewer medical conditions [24]. Of note, the CCI does not assess for hypertension which may impact incontinence symptoms due to antihypertensive drugs, particularly diuretics [28]. Currently, most of the published data regarding chronic disease and incontinence focuses on the metabolic syndrome, and there appears to be a link between the number of metabolic syndrome components, including obesity, diabetes, dyslipidemia, and hypertension and severity of LUTS. In addition, although the association between diabetes and UUI has been demonstrated, women with diabetes and metabolic syndrome had a higher prevalence of LUTS and overactive bladder [29]. However, there was no association between metabolic syndrome and International Prostate Symptom Scores in men and women in another study [30]. It is currently not well understood whether there is any causality of incontinence from abnormalities related to metabolic syndrome or other chronic disease states. 

 The strengths of this study were its multicenter design and inclusion of a large population of well-characterized women undergoing SUI surgery. The use of validated instruments and standardized anatomic characterization further strengthens our findings. The main limitation of our analysis is the lack of generalizability to all women with SUI; therefore, our findings cannot be applied to women with urge predominant symptoms or who are not undergoing surgery. However, our study population remains of particular interest given that greater bothersome baseline urgency symptoms have been consistently shown to predict patient dissatisfaction in women undergoing SUI surgery. As over 200,000 women a year undergo SUI surgery in the United States alone [31], it is important to identify opportunities to modify these symptoms in the hopes of improving patient outcomes.

## 5. Conclusions

 Although we did not find any baseline patient variables that were associated with UUI in patients undergoing SUI surgery, interesting questions regarding the effect of systemic medical conditions on overactive bladder symptoms were raised and may represent targets for intervention. Why is depression associated with more severe UUI? Does UUI in a patient with depression represent a different pathophysiologic condition than a patient with a more “localized” pelvic floor disorder? Does the association of number and/or severity of chronic diseases with worsening of UUI/LUTS indicate a more systemic problem affecting bladder function that is less likely to resolve after SUI surgery? Clearly, further investigation using a well-characterized population and validated pelvic floor instruments is necessary to clarify the role of depression and concomitant medical conditions in the pathophysiology of UUI and LUTS in women. In addition, greater emphasis on “phenotyping” of our patient populations may shed light on the complex relationship between UUI and SUI and allow clinicians to more accurately predict surgical outcomes.

## Figures and Tables

**Table 1:** Descriptive statistics by UUI status and results of separate bivariate logistic regression models with outcome of those having UUI. Results reported separately for the SISTEr and TOMUS trials.

Covariate	SISTEr (*N* = 651)				TOMUS (*N* = 593)			
	Yes (*n* = 455)	No (*n* = 196)	*P* value	OR (95% CI)	Yes (*n* = 403)	No (*n* = 190)	*P* value	OR (95% CI)
Menopausal status/hormone replacement therapy			0.002				0.01	
No HRT, Postmenopausal	178 (39%)	53 (27%)		1	169 (42%)	74 (39%)		1
Yes HRT, Postmenopausal	156 (34%)	67 (34%)		0.69 (0.46–1.05)	126 (31%)	44 (23%)		1.25 (0.81–1.94)
Premenopausal	120 (26%)	76 (39%)		0.47 (0.31–0.72)	106 (26%)	72 (38%)		0.64 (0.43–0.97)
Prior to UI surgery and/or treatment			0.01				0.19	
No	206 (45%)	110 (56%)			165 (41%)	89 (47%)		
Yes	249 (55%)	86 (44%)		1.55 (1.10–2.17)	236 (59%)	101 (53%)		1.26 (0.89–1.78)
Prior to hysterectomy			0.09				0.03	
No	306 (67%)	145 (74%)			276 (69%)	147 (77%)		
Yes	149 (33%)	51 (26%)		1.38 (0.95–2.01)	125 (31%)	43 (23%)		1.55 (1.04–2.31)
Race/ethnicity			0.16				0.06	
Hispanic	56 (12%)	15 (8%)		1.79 (0.98–3.27)	53 (13%)	17 (9%)		1.66 (0.93–2.95)
Non-Hispanic white	323 (71%)	155 (79%)		1	307 (76%)	163 (86%)		1
Non-Hispanic black	34 (7%)	10 (5%)		1.63 (0.79–3.39)	14 (3%)	3 (2%)		2.48 (0.70–8.75)
Non-Hispanic other	41 (9%)	16 (8%)		1.23 (0.67–2.26)	29 (7%)	7 (4%)		2.20 (0.94–5.13)
Parity			0.68				0.48	
No pregnancies	16 (5%)	6 (4%)		1	17 (6%)	8 (5%)		1
One pregnancy	25 (7%)	14 (10%)		0.67 (0.21–2.10)	37 (12%)	27 (18%)		0.64 (0.24–1.71)
Two pregnancies	112 (33%)	49 (36%)		0.86 (0.32–2.32)	111 (37%)	51 (34%)		1.02 (0.42–2.53)
Three or more pregnancies	185 (55%)	69 (50%)		1.01 (0.38–2.67)	133 (45%)	65 (43%)		0.96 (0.39–2.35)
Education			0.002				<.0001	
	48 (11%)	6 (3%)		1	25 (6%)	8 (4%)		1
High school/GED	115 (25%)	56 (29%)		0.26 (0.10–0.64)	109 (27%)	39 (21%)		0.89 (0.37–2.15)
	188 (41%)	70 (36%)		0.34 (0.14–0.82)	160 (40%)	57 (30%)		0.90 (0.38–2.11)
BA/BS	56 (12%)	42 (21%)		0.17 (0.07–0.43)	49 (12%)	52 (27%)		0.30 (0.12–0.73)
Grad/Prof	48 (11%)	22 (11%)		0.27 (0.10–0.73)	60 (15%)	34 (18%)		0.56 (0.23–1.39)
Prolapse stage			1				0.34	
Stage 0 or 1	110 (24%)	48 (24%)		1	189 (47%)	77 (41%)		1
Stage 2	271 (60%)	116 (59%)		1.02 (0.68–1.53)	183 (45%)	96 (51%)		0.78 (0.54–1.12)
Stage 3+	74 (16%)	32 (16%)		1.01 (0.59–1.72)	31 (8%)	17 (9%)		0.74 (0.39–1.42)
UI Severity (PGI-S)*	N/A						0.001	
Normal					38 (9%)	23 (12%)		1
Mild					30 (7%)	30 (16%)		0.61 (0.29–1.25)
Moderate					202 (50%)	97 (51%)		1.26 (0.71–2.23)
Severe					132 (33%)	39 (21%)		2.05 (1.09–3.84)
Depression (PHQ-9)*	N/A						0.0001	
<10					293 (74%)	165 (88%)		1
10+ (defined as depression)					103 (26%)	22 (12%)		2.64 (1.60–4.34)
Charlson Comorbidity Index (CCI)*	N/A						0.03	
0					287 (71%)	153 (81%)		1
1					69 (17%)	27 (14%)		1.36 (0.84–2.22)
2+					47 (12%)	10 (5%)		2.51 (1.23–5.10)

	Mean (SD)	Mean (SD)			Mean (SD)	Mean (SD)		

UDI stress score (UDI-S)	79.8 (20.2)	73.8 (25.0)	0.002	1.13 (1.05–1.22)	75.4 (20.6)	72.5 (23.1)	0.13	1.06 (0.98–1.15)
MESA stress score	20.2 (4.1)	17.2 (5.0)	<0.0001	1.15 (1.11–1.20)	20.0 (4.2)	17.9 (5.0)	<.0001	1.11 (1.06–1.15)
Age (years)	52.2 (10.1)	51.4 (10.8)	0.37	1.08 (0.92–1.27)	53.6 (10.9)	51.3 (10.9)	0.02	1.21 (1.03–1.43)
Body mass index	30.4 (6.3)	28.9 (5.4)	0.003	1.05 (1.02–1.08)	30.7 (7.0)	29.5 (6.2)	0.03	1.03 (1.00–1.06)
IIQ total	190.7 (100.7)	126.3 (87.7)	<0.0001	1.07 (1.05–1.09)	169.4 (98.2)	113.4 (83.4)	<.0001	1.07 (1.05–1.09)
Pad weight (g)	48.6 (87.6)	31.3 (54.1)	0.01	1.04 (1.01–1.08)	38.1 (66.9)	31.5 (66.6)	0.27	1.02 (0.99–1.05)

*UI severity, depression, and number of comorbidities were not collected in the SISTEr trial.

**Table 2:** Final multivariable linear regression models for the SISTEr and TOMUS trials with outcome of UDI-I.

Covariate	SISTEr		TOMUS	
	*P* value	Estimate**	*P* value	Estimate**
Race/ethnicity	0.001		0.18	
Hispanic		41.30		40.46
Non-Hispanic white		48.17		41.24
Non-Hispanic black		55.67		47.06
Non-Hispanic other		52.65		47.80
Education	0.006		<0.0001	
<High school		56.23		46.50
High school/GED		50.19		50.52
>High school		49.04		45.27
BA/BS		42.90		38.20
Grad/Prof		48.87		40.20
UI Severity (PGI-S)*			<0.0001	.
Normal	N/A			42.11
Mild	N/A			35.08
Moderate	N/A			45.57
Severe	N/A			53.80
Charlson Comorbidity Index (CCI)*			0.0003	
0	N/A			39.35
1	N/A			42.11
2+	N/A			50.97
UDI stress score	<0.0001	0.17	0.06	0.08
Body mass index	0.002	0.44	0.34	0.13
IIQ total	<0.0001	0.11	<0.0001	0.09
Pad weight (10 g per unit)	0.03	0.23	0.004	0. 39

*UI severity and number of comorbidities were not collected in the SISTEr trial.

**For categorical predictors, least squares means are reported, and for continuous covariates, regression coefficients are reported.

**Table 3:** Final multivariable linear regression models for the SISTEr and TOMUS trials with outcome urge symptoms from the MESA scale.

Covariate	SISTEr		TOMUS	
	*P* value	Estimate**	*P* value	Estimate**
Education	<0.0001		0.002	
<High school		8.21		7.12
High school/GED		6.50		7.15
>High school		6.52		6.65
BA/BS		5.34		5.62
Grad/Prof		6.48		5.90
Depression (PHQ-9)*	N/A		0.01	
<10				6.06
10+ depression				6.91
MESA stress score	<0.0001	0.41	<0.0001	0.41
Age (10 years per unit)	0.001	0.42	<0.0001	0.54
Pad weight (10 g per unit)	0.001	0.05	0.0007	0.07

*Depression information was not collected in the SISTEr trial.

**For categorical covariates, least squares means are reported, and for continuous covariates, regression coefficients are reported.
